# Primary renal leiomyosarcoma with a tumor thrombus in the inferior vena cava

**DOI:** 10.1002/iju5.12396

**Published:** 2021-11-19

**Authors:** Mikio Konno, Takahiro Osawa, Kiyohiko Hotta, Ai Shimizu, Takashige Abe, Ryuji Matsumoto, Hiroshi Kikuchi, Nobuo Shinohara

**Affiliations:** ^1^ Department of Urology Hokkaido University Hospital Sapporo Japan; ^2^ Department of Surgical Pathology Hokkaido University Hospital Sapporo Japan

**Keywords:** kidney, leiomyosarcoma, thrombus, vena cava

## Abstract

**Introduction:**

We report a rare case of primary renal leiomyosarcoma with a tumor thrombus in the inferior vena cava.

**Case presentation:**

A 54‐year‐old woman presented with right flank pain and abdominal distension. Physical examination findings were unremarkable. Abdominal computed tomography revealed a heterogeneously enhancing right solid renal mass with a thrombus in the renal vein extending into the inferior vena cava. Magnetic resonance imaging demonstrated a renal tumor with a thrombus about 4 cm below the hepatic vein. Chest computed tomography and bone scintigraphy were negative. The patient underwent right radical nephrectomy and vena cava thrombectomy. Histophathologic evaluation of the resected tumor confirmed the diagnosis of leiomyosarcoma. She underwent no adjuvant therapy. Seven months after surgery, the patient died following a 2‐month history of multiple pulmonary and hepatic metastases.

**Conclusion:**

This report highlights the importance of considering the possibility of renal leiomyosarcoma invasion to the inferior vena cava, similar to renal cell carcinoma.

**Abbreviations & Acronyms**
CT: computed tomography
HPF: high power field
IVC: inferior vena cava
MSI‐H: microsatellite instability‐high
RCC: renal cell carcinoma
STS: soft tissue sarcoma

**Keynote message**

We report a rare case of primary renal leiomyosarcoma with a tumor thrombus in the inferior vena cava (IVC). Similar to other retroperitoneal sarcomas, complete surgical resection is the most effective treatment. This case report highlights the importance of considering the rare possibility for cancer types other than renal cell carcinoma to extend into the IVC.

## Introduction

While sarcomas constitute only about 1 to 2% of primary renal tumors, these are known to have poor prognosis. Among these, leiomyosarcoma is the most common type, composing approximately 50–60% of the total incidence of renal sarcomas.^1^ Although RCC is the most common primary tumor that involves the IVC, other malignancies have been reported to cause tumor thrombus in the IVC.^2^, ^3^

Here, we report a case of primary renal leiomyosarcoma with a tumor thrombus in the IVC and review related cases in the literature.

## Case presentation

A previously healthy 54‐year‐old woman presented with right flank pain and abdominal distension. Physical examination findings were unremarkable. Blood test showed a decreased level of hemoglobin (9.1g/dl: normal >11.6) and an increased level of lactic dehydrogenase (640 IU/ml; normal <460) and C‐reactive protein (0.67mg/l; normal <0.24). Urinalysis demonstrated elevated red blood cells [5–9 cells per HPF] and white blood cells (10–19 cells per HPF).

**Fig. 1:**
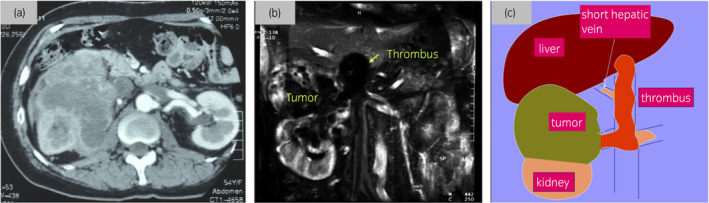
(a) Abdominal computerized tomography (CT) revealed a heterogeneously enhancing solid mass in the right kidney and a thrombus in the IVC. (b) Magnetic resonance imaging (T2‐weighted) reveals tumor of slightly high signal intensity in the right kidney and thrombus extending into renal vein and vena cava. (c) Shema of renal tumor and vena cava thrombus. The thrombus extended in short hepatic vein and left renal vein

Abdominal CT revealed a heterogeneously enhancing solid mass in the right kidney with a thrombus extending from the renal vein to the IVC (Fig. 1a). Magnetic resonance imaging showed a renal tumor with a level II thrombus 4 cm below the hepatic vein (Fig. 1b). Chest CT and bone scintigraphy findings were unremarkable. These findings were consistent with a preoperative diagnosis of right RCC with an IVC thrombus.

The patient underwent right radical nephrectomy with vena cava thrombectomy. The skin was incised through a chevron incision and a combined upper midline incision. The tumor, IVC, and left renal vein were exposed using a cocker maneuver. Several short hepatic veins were ligated and dissected, and the liver was mobilized. A thrombus found in one of the short hepatic veins was removed (Fig. 1c). IVC was secured above the thrombus and below the hepatic vein, which was clamped along with the left renal vein. An incision was made in front of the IVC and its right wall was partially attached to remove the tumorous thrombus as a mass with the right renal tumor. Fortunately, no enlarged lymph nodes were identified. Total operation time was 11 h and 6 min. Total blood loss was 1006 ml and 5 units of packed red blood cells were transfused.

**Fig. 2:**
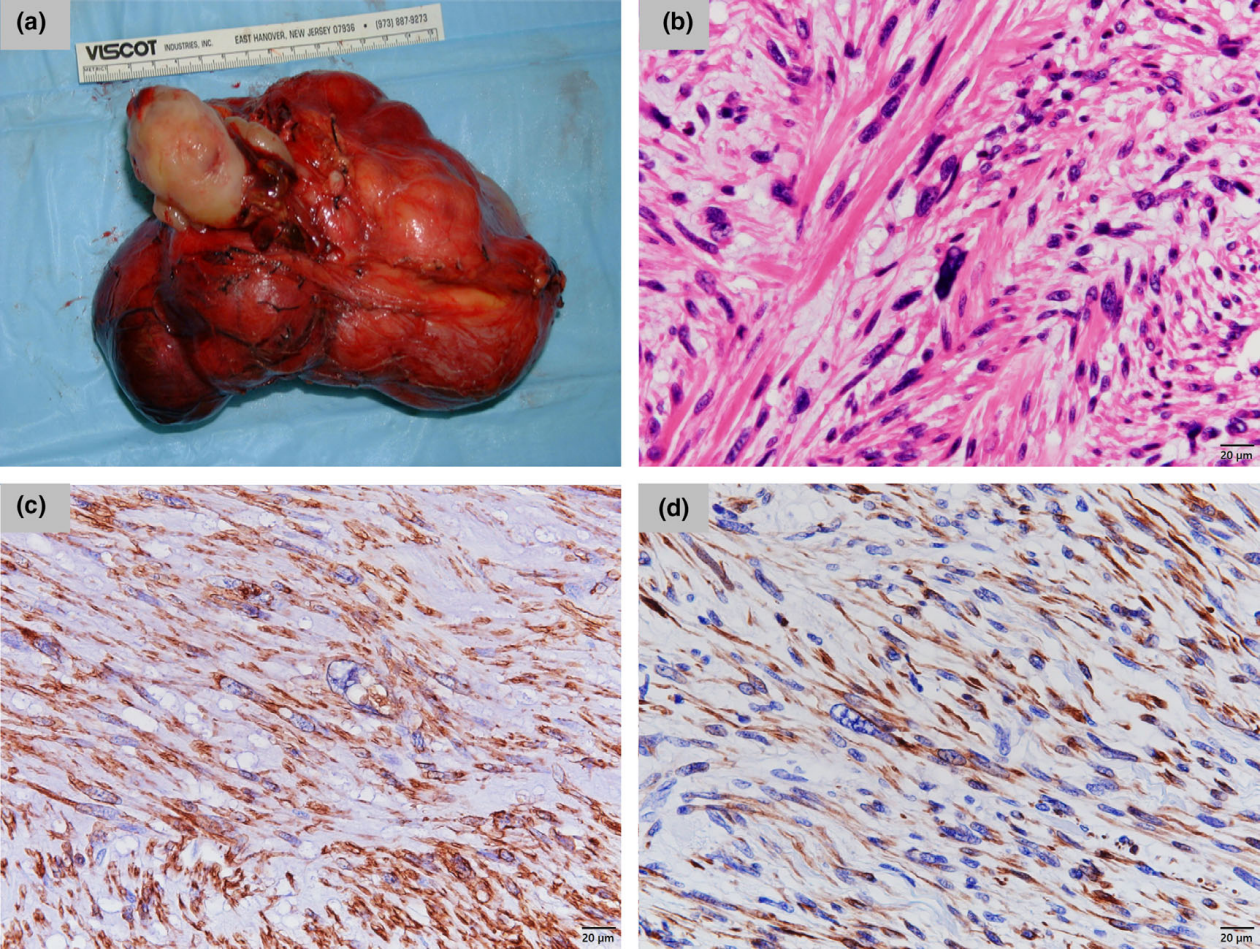
(a) Gross specimen with gray and film tumor of the right kidney and clearly encapsulized tumor thrombus. (b) The tumor and thrombus are composed of spindle‐shaped cells forming tightly interwoven fascicles. (HE. ×400) (c, d) Immunochemistry shows that the spindle cells are reactive for α‐smooth muscle actin (c) and desmin (d). (×400)

Gross examination revealed a gray‐to‐white well‐circumscribed renal mass and clearly encapsulized tumor thrombus. (Fig. 2a). Histopathologic examination demonstrated spindle‐shaped cells with elongated hyperchromatic nuclei and moderate pleomorphism (Fig. 2b). Immunochemistry showed that the spindle cells were reactive for α‐smooth muscle actin (Fig. 2c) and desmin (Fig. 2d). These findings were consistent with a definitive diagnosis of renal leiomyosarcoma with a tumor thrombus in the IVC. Because surgical extirpation was performed on both the mass and the thrombus, adjuvant therapy was not initiated. However, 5 months after surgery, she presented with multiple pulmonary and hepatic metastases. We offered her the option of systemic treatment, but she chose the best supportive care. She died of the disease 7 months after surgery.

## Discussion

Primary renal sarcoma in adult is a rare tumor type and contribute only about 1 to 2% of all primary renal tumors. Among renal sarcomas, leiomyosarcoma is the most common type, accounting for 50–60% of cases.^1^ In general, patients with renal sarcoma have a worse prognosis than patients with other urinary tract sacromas.^4^ RCC is the most common primary tumor with IVC involvement. However, other malignancies (i.e., urothelial carcinomas, adrenal tumors, testicular carcinomas, and hepatocellular carcinoma) have been reported to cause IVC tumor thrombi.^2^, ^3^ To the best of our knowledge, there is only one other report of renal leiomyosarcoma with IVC thrombus in a Japanese patient.^5^

Most cases of renal leiomyosarcoma remain unrecognized until the mass significantly enlarges, as in this case.^5^ Imaging studies are useful for surgical planning and detection of metastasis. However, no radiologic feature is specific to leiomyosarcoma, marking preoperative diagnosis extremely challenging.^4^, ^5^ In our case, although RCC was suspected preoperatively, histopathologic examination of resected tumor revealed leiomyosarcoma. This suggests that pathologic evaluation is crucial for the diagnosis of leiomyosarcoma. Immunohistochemistry may be important in confirming the diagnosis by demonstrating the reactively of spindle cells for α‐smooth muscle actin, desmin, and h‐caldesmon.^6^ In our case, the positive immunostaining results for α‐smooth muscle actin and desmin verified the diagnosis.

Surgery with a negative margin is a preferred strategy for treatment in all patients with localized STS, as well as in patients with renal leiomyosarcomas.^6^ In general, adjuvant/neoadjuvant chemotherapy and/or radiotherapy do not contribute to improved survival in patients with leiomyosarcoma.^4^ Adjuvant radiotherapy is often not feasible due to safety concerns to normal tissue in the resection bed. Regarding perioperative chemotherapy, most clinical trials evaluating adjuvant chemotherapy include patients with many histologic types, making it difficult to formulate specific recommendations for each histologic type.^6^ In this case, complete resection without adjuvant therapy was done.

The French Federation of Cancer Centers Sarcoma Group System is the most commonly used grading system for adult sarcomas based on tumor differentiation, mitotic count, and tumor necrosis. However, there is no evidence showing the correlation between histologic features and tumor progression in patients with renal leiomyosarcoma.^7^, ^8^ In our case, histopathologic evaluation revealed a total differentiation score of 2, a mitotic count score of 3, and a necrosis score of 1. These findings imply that the tumor had a histologic grade of 3, which had the worse prognosis in the system. This was consistent with the findings of our case because the patient died due to progressive pulmonary and hepatic metastases a few months after surgery.

For the patients with unresectable or recurrent STS, single‐agent doxorubicin is the standard systemic treatment. However, compared to other pathologic types such as synovial sarcoma and liposarcoma, the response rate (10 to 25%) for leiomyosarcoma was worse.^9^ In Japan, three anticancer drugs (i.e., pazopanib, trabectedin, and eribulin) have been approved since 2012 as second‐line or later treatment for patients with advanced STS.^10^ Phase 3 trials have shown the efficacy of these drugs in the survival of patients with leiomyosarcoma.^11^, ^12^, ^13^ In our case, initiation of these drugs could have altered the patient’s clinical outcomes. Although we did not have such option to perform the currently available genome profiling test for this case, such a test might have found other effective treatment options. Wang YJ et al. recently reported that they introduced pembrolizumab to a patient with MSI‐H uterine leiomyosarcoma.^14^ The programmed cell death‐1 inhibitor did not improve the overall survival of the patients; however, the functional status and quality‐of‐life showed massive improvement. Despite the rarity of MSI‐H status in patients with leiomyosarcoma, the test for MSI‐H should be considered for these patients to widen the treatment option.^15^

## Conclusion

We have described a rare case of primary renal leiomyosarcoma with an IVC thrombus. Similar to RCC, this study highlights the importance of considering the rare possibility of renal leiomyosarcoma invasion to the renal vein and IVC. Despite the risks involved during resection of the IVC thrombus, we emphasize the crucial role of complete surgical resection in improving patient outcomes.

## Conflict of interest

The authors declare no conflict of interest.

## Approval of the research protocol by an Institutional Reviewer Board

Not applicable.

## Informed consent

We obtained informed consent from the patient.

## Registry and the registration no. of the study/trial

Not applicable.
